# Accuracy of genomic prediction for growth and carcass traits in Chinese triple-yellow chickens

**DOI:** 10.1186/s12863-014-0110-y

**Published:** 2014-10-15

**Authors:** Tianfei Liu, Hao Qu, Chenglong Luo, Dingming Shu, Jie Wang, Mogens Sandø Lund, Guosheng Su

**Affiliations:** Institute of Animal Science, Guangdong Academy of Agricultural Sciences, Guangzhou, 510640 China; Department of Molecular Biology and Genetics, Center for Quantitative Genetics and Genomics, Aarhus University, DK-8830, Tjele, Denmark; State Key Laboratory of Livestock and Poultry Breeding, Guangzhou, 510640 China

**Keywords:** Accuracy, Breeding value, Cross-validation, Genomic prediction

## Abstract

**Background:**

Growth and carcass traits are very important traits for broiler chickens. However, carcass traits can only be measured postmortem. Genomic selection may be a powerful tool for such traits because of its accurate prediction of breeding values of animals without own phenotypic information. This study investigated the efficiency of genomic prediction in Chinese triple-yellow chickens. As a new line, Chinese triple-yellow chicken was developed by cross-breeding and had a small effective population. Two growth traits and three carcass traits were analyzed: body weight at 6 weeks, body weight at 12 weeks, eviscerating percentage, breast muscle percentage and leg muscle percentage.

**Results:**

Genomic prediction was assessed using a 4-fold cross-validation procedure for two validation scenarios. In the first scenario, each test data set comprised two half-sib families (family sample) and the rest represented the reference data. In the second scenario, the whole data were randomly divided into four subsets (random sample). In each fold of validation, one subset was used as the test data and the others as the reference data in each single validation. Genomic breeding values were predicted using a genomic best linear unbiased prediction model, a Bayesian least absolute shrinkage and selection operator model, and a Bayesian mixture model with four distributions. The accuracy of genomic estimated breeding value (GEBV) was measured as the correlation between GEBV and the corrected phenotypic value. Using the three models, the correlations ranged from 0.448 to 0.468 for the two growth traits and from 0.176 to 0.255 for the three carcass traits in the family sample scenario, and were between 0.487 and 0.536 for growth traits and between 0.312 and 0.430 for carcass traits in the random sample scenario. The differences in the prediction accuracies between the three models were very small; the Bayesian mixture model was slightly more accurate. According to the results from the random sample scenario, the accuracy of GEBV was 0.197 higher than the conventional pedigree index, averaged over the five traits.

**Conclusions:**

The results indicated that genomic selection could greatly improve the accuracy of selection in chickens, compared with conventional selection. Genomic selection for growth and carcass traits in broiler chickens is promising.

**Electronic supplementary material:**

The online version of this article (doi:10.1186/s12863-014-0110-y) contains supplementary material, which is available to authorized users.

## Background

Genomic selection has been widely applied in dairy cattle breeding [1-5] and has started to be used in other livestock species [6-8]. The Illumina Chicken 60K SNP Beadchip is available [9]; therefore, genomic selection has been taken into consideration in chicken breeding. Genomic selection makes it possible to select both genders accurately at an early stage of life. An analysis of the economic efficiency of genomic selection in dairy cattle, carried out by Schaeffer [10], showed that genomic selection can increase genetic gain per year by a factor of two, while saving up to 90% of the cost for maintaining bulls, compared with a conventional breeding scheme. However, the large benefit of genomic selection for dairy cattle is mainly caused by a large reduction of generation interval. This would not be the case for other animals, such as broilers. On the other hand, chicken breeding may benefit from increasing the accuracy of genomic selection, especially for traits that are difficult or costly to measure.

Carcass traits are very important traits for broiler chickens. However, carcass traits cannot be directly measured from breeding candidates. Therefore, indirect measures are usually used as indicator traits, for example, using ultrasound measurement of breast meat to predict actual weight of breast meat. Breeding values of carcass traits predicted using the indirect measurements could be inaccurate. With genome-wide dense markers, it is possible to predict breeding values accurately, based on direct measurements of carcass traits from a reference population. Recently, some studies on genomic selection in chickens have been carried out [11-13]; however, none of these studies dealt with direct measures of carcass traits.

In general, Chinese local breeds, are characterized by high meat quality and rich flavor, which meet popular demand in China. Major breeds of Chinese local chickens have yellow beak, yellow feather and yellow claw, and therefore they are named Chinese triple-yellow chickens. However, slow growth is another characteristic of Chinese local breeds. Cross-breeding is often used to breed synthetic lines in chickens [14,15]. In the current study, a new crossbred line with fast growth, high meat quality and rich flavor was bred from a local line and a commercial line. Crossbred lines usually originate from a small number of founder birds. The new lines have few phenotypic records, making it difficult to obtain highly accurate EBV using conventional best linear unbiased prediction (BLUP).

The accuracy of genomic prediction is key to the successful application of genomic selection. A number of statistical models have been proposed to predict genomic values [16-21], among which genomic best linear unbiased prediction (GBLUP) models and Bayesian variable selection or variable shrinkage models have been widely used. The main differences between these models are assumptions concerning distributions of genomic marker effects. GBLUP models assume that effects of all markers are normally distributed with the same variance [17]. Bayesian variable shrinkage and selection models assume that variances of marker effects are different of which the Bayesian least absolute shrinkage and selection operator (BayesLASSO) assumes that effects of all markers follow a double exponential distribution [18], and Bayesian mixture models assume that most markers have null or very small effects, and a small number of markers have large or moderate effects [2,16,22,23].

Accuracy of genomic prediction is greatly dependent on the size of reference population and effective population size. Larger reference population provides more information of phenotypic records , and smaller effective population size leads to smaller effective number of loci [24]. Therefore larger reference population and small effective population size would give higher accuracy of genomic prediction [24-26]. However, for some newly established lines which are originated from a small number of foundation animals, both the effective population size and reference population sizes are usually small. An important question is whether such population can get benefit from genomic selection.

The main objective of this study was to investigate the accuracy of genomic selection for a population with small effective population size but also small reference population. Genomic prediction was performed for two growth traits and three carcass traits, using GBLUP, BayesLASSO and Bayesian mixture models, based on data from a Chinese triple-yellow chicken population, which had a small number of foundation animals and only had records from the F_2_ generation.

## Results

Estimates of genetic parameters based on the full phenotypic data (all records used) are presented in Table 1. Estimates of heritability (proportion of additive genetic variance to phenotypic variance) were 0.26 and 0.13 for body weight at the 6^th^ week (BW6) and at the 12^th^ week (BW12), and were 0.44, 0.39 and 0.39 for eviscerating percentage (EP), breast muscle percentage (BMP) and leg muscle percentage (LMP), respectively.

**Table 1:** **Estimates of genetic parameters using a model incorporating a pedigree**-**based relationship matrix and based on the full data**

**Trait** ^**1**^	${\boldsymbol{\upsigma}}_{\mathbf{a}}^{\mathbf{2}}$	${\boldsymbol{\upsigma}}_{\mathbf{a}}^{\mathbf{2}}$	***h*** ^***2***^
BW6	3044.50	8579.90	0.26 ± 0.13
BW12	10433.86	67520.65	0.13 ± 0.09
EP	1.38	1.76	0.44 ± 0.14
BMP	0.83	1.29	0.39 ± 0.14
LMP	0.90	1.41	0.39 ± 0.13

^1^ *BW*6 = body weight at 6th weeks; *BW*12 = body weight at 12th weeks; *EP* = eviscerating percentage; *BMP* = breast muscle percentage; *LMP* = leg muscle percentage.

Table 2 shows the correlations between corrected phenotypic values and the predictions for the birds based on the data pooled over the four folds of validations, more results in each fold are presented in Additional file 1: Table S1. The correlations in the validation scenario of family sampling (ValFamily) reflected the accuracies of predictions for the candidates when neither they nor their half/full sibs have phenotypic records. In contrast, the correlations in the validation scenario of random sampling (ValRandom) reflected the accuracies of predictions for the candidates without records; however, their half/full sibs may have phenotypic records.

**Table 2:** **Correlations between corrected phenotypic values and genomic predictions for the birds in the test sets assessed by cross**-**validation**

**Trait** ^**1**^	**BLUP**	**GBLUP**	**BayesLASSO**	**BayesMix4**
Family sample				
BW6	−0.078^a^	0.448^b^	0.449^b^	0.463^b^
BW12	0.014^a^	0.449^b^	0.452^b^	0.468^b^
EP	−0.009^a^	0.252^b^	0.255^b^	0.239^b^
BMP	0.092^a^	0.251^a^	0.251^a^	0.254^a^
LMP	0.152^a^	0.188^a^	0.183^a^	0.176^a^
Mean	0.034	0.318	0.318	0.320
Random sample				
BW6	0.194^a^	0.525^b^	0.525^b^	0.536^c^
BW12	0.107^a^	0.487^b^	0.487^b^	0.506^c^
EP	0.313^a^	0.395^c^	0.394^c^	0.390^b^
BMP	0.237^a^	0.313^b^	0.312^b^	0.333^c^
LMP	0.326^a^	0.430^c^	0.430^c^	0.428^b^
Mean	0.236	0.430	0.430	0.439

^1^ *BW*6 = body weight at 6th weeks; *BW*12 = body weight at 12th weeks; *EP* = eviscerating percentage; *BMP* = breast muscle percentage; *LMP* = leg muscle percentage.

^a-c^Within a row, estimates without a common superscript differ significantly (P < 0.05), according to paired t test.

As shown in Table 2, the accuracies of the predictions in ValFamily were lower than those in ValRandom for all traits. Moreover, the accuracies of genomic estimated breeding value (GEBV) were much higher than those of conventional EBV. The superiority of genomic prediction over conventional prediction was more pronounced in ValFamily, where the accuracies of EBV were close to zero for most traits because there was no information from close relatives of the candidates. Averaged over the five traits, the accuracy of EBV was 0.034 and the accuracy of GEBV from GBLUP was 0.318 in ValFamily. The accuracies were 0.236 and 0.430 for EBV and GEBV, respectively, in ValRandom.

In general, GEBV predicted by GBLUP and BayesLASSO had similar accuracies (Table 2), while BayesianMix4 predict GEBV slightly more accurate than GBLUP and BayesLASSO for BW6, BW12 and BMP while less accurate for LMP in the ValRandom scenario. Averaged over the five traits, the accuracies were 0.318, 0.318 and 0.320 for GBLUP, BayesLASSO and BayesMix4, respectively, in the ValFamily scenario, and were 0.430, 0.430 and 0.439 in the ValRandom scenario.

The statistic power of the accuracies from the three genomic prediction models were acceptable (P > 0.85) for all five traits in both validation scenarios. But the statistic powers of the accuracies from the conventional model were unacceptable (P < 0.85) for all traits in the ValFamily scenario. The differences between the three genomic prediction models and conventional model were significant (P < 0.05) for all traits in the ValRandom scenario, and were significant for BW6, BW12 and EP in the ValFamily scenario. Although the differences between accuracies of predictions from conventional and genomic prediction models were larger in ValFamily scenario than in ValRandom scenario, the differences for BMP and LMP were not statistically significant in ValFamily scenario. This could be resulted from larger sampling error in ValFamily scenario. In this scenario, the individual within a fold are strongly related, consequently reducing effective sample size and increasing sampling error. It was also reflected by a large variation in correlations between folds in ValFamily scenario (Additional file 1: Table S1). The differences between the three genomic prediction models were not significant for all traits in the ValFamily scenario. The differences between GBLUP and BayesLASSO were not significant in the ValRandom scenario.

The accuracies of the genomic predictions based on the full phenotypic data were also investigated. As shown in Table 3, the correlations between GEBV and corrected phenotypic values were higher than those between EBV and the corrected phenotypic values for BW6, BW12 and LMP. This indicated that genomic prediction was more accurate than conventional pedigree-based prediction for the three traits, even in cases where the candidates had their own phenotypic records. The correlations between GEBV and the corrected phenotypic values were lower than those between EBV and the corrected phenotypic values for EP and BMP. However, the correlations for carcass traits were less meaningful in practical breeding because these traits cannot be directly measured in breeding candidates.

**Table 3:** **Correlations between corrected phenotypic values and predictions obtained from the analysis of the full data**

**Trait** ^**1**^	**BLUP**	**GBLUP**	**BayesLASSO**	**BayesMix4**
BW6	0.772	0.908	0.905	0.891
BW12	0.643	0.896	0.893	0.873
EP	0.872	0.813	0.814	0.837
BMP	0.866	0.789	0.795	0.808
LMP	0.838	0.876	0.878	0.889
Mean	0.798	0.857	0.857	0.860

^1^ *BW*6 = body weight at 6th weeks; *BW*12 = body weight at 12th weeks; *EP* = eviscerating percentage; *BMP* = breast muscle percentage; *LMP* = leg muscle percentage.

As shown in Table 4, in ValFamily, the regression coefficients for LMP were far from one for all models (ranging from 0.506 to 0.595), but close to one for the other four traits. In ValRandom, the regression coefficients for all traits were close to one. The regression coefficients were almost identical for GBLUP and BayesLASSO in the two scenarios. Compared with these models, BayesMix4 led to genomic predictions with less bias for BW6 and BW12, but genomic predictions with larger biases for the other three traits.

**Table 4:** **Regression coefficients of the corrected phenotypic values for the genomic predictions of the birds in the test sets during cross**-**validation**

**Trait** ^**1**^	**BLUP**	**GBLUP**	**BayesLASSO**	**BayesMix4**
Family sample				
BW6	−1.064	1.079	1.078	1.007
BW12	0.265	1.186	1.194	1.071
EP	−0.080	1.027	1.022	0.893
BMP	0.507	1.034	1.005	0.819
LMP	1.424	0.595	0.569	0.506
Mean Dev.^2^	0.959	0.146	0.146	0.172
Random sample				
BW6	0.845	1.045	1.046	1.015
BW12	0.686	1.054	1.054	1.008
EP	0.914	0.964	0.956	0.890
BMP	0.896	0.961	0.948	0.863
LMP	0.971	0.955	0.953	0.897
Mean Dev.^2^	0.138	0.044	0.049	0.075

^1^ *BW*6 = body weight at 6th weeks; *BW*12 = body weight at 12th weeks; *EP* = eviscerating percentage; *BMP* = breast muscle percentage; *LMP* = leg muscle percentage.

^2^Mean of absolute deviation from 1 for regression coefficient.

## Discussion

### The accuracy of genomic prediction

This study investigated the performance of genomic predictions in Chinese triple-yellow chickens, using a reference population of F_2_ birds. Four-fold cross-validation showed that, averaged over two growth traits and three carcass traits, the accuracy of GEBV was about 0.285 higher than EBV in ValFamily, and 0.197 higher than EBV in ValRandom. For LMP and the two growth traits, even for the birds that had their own records, the accuracy of GEBV was higher than that of EBV.

A number of previous studies have reported that genomic evaluation is more accurate than conventional genetic evaluation, for example in dairy cattle [2,5,27], beef cattle [28-30], pigs [6,31,32] and sheep [8,33]. In chickens, genomic evaluation increased accuracies by up to 100% for selection at an early age and by up to 88% for selection at a later age for egg production in layer chickens [34]. The accuracy of GEBV was almost 50% higher than EBV for ultrasound measurement of breast meat in broiler chickens [13].

The current study was based on the data from F_2_ birds from a newly established line with a small base population; therefore, the number of phenotypic records was small. Although the training data in the cross-validation was small (381–385 birds), the results showed that the accuracies of the predictions were relatively high, considering the size of the training data. This could be attributed to the small effective population size in this line. If the effective population size is small, it is expected that animals will share larger chromosome segments and the genomic predictions will be more accurate. The influence of the effective population size on the accuracy of genomic prediction has been demonstrated in some studies [24,26]. In addition, the data in this study came from F2 population. The F2 population is a structured population where chromosomes are inherited in large segments. Genomic prediction in F2 population would be more accurate than outbred populations due to long-range linkage disequilibrium. The results of the present study indicate that for a new line originating from a small number of foundation birds from genetically different origins, genomic selection would be much better than conventional selection.

Genomic selection greatly benefits the traits that are difficult to be measured or cannot be directly measured when the animals are selected. As shown in this study, the gain from genomic prediction was relatively small when the animal had own records (Table 3), while relatively large when the animal did not have own records (Table 2). In chicken, birds have got records of growth but do not have records of carcass traits at the time of selection. Therefore, the benefit from genomic prediction would be larger for carcass traits than for growth traits.

Almost all the regression coefficients of corrected phenotypic values on GEBV were close to one in both ValFamily and ValRandom, except the ones for LMP in ValFamily which were far from one for all models, indicating a serious inflation of GEBV for LMP in this scenario. The reason for this unexpected regression coefficient is not clear. One possible reason could be that markers may fit part of noise, consequently reducing accuracy of GEBV (lowest for LMP in ValFamily scenario) and regression coefficient (inflating variance of GEBV). An inflation of GEBV is common phenomenon in genomic prediction [27,35] caused by a number of factors. It needs further studies to detect the real reason for the inflation of GEBV for LMP in ValFamily scenario.

### Relationship between the predicted animals and the training animals

One of the factors influencing the accuracy of genomic prediction is the relatedness between the predicted animals and the training animals. In most studies [5,7,12,36] of the accuracy of genomic prediction, validation is usually performed by taking younger animals as the test data, because this is consistent with the real life scenario: parents have performance records, but the candidates only have genotype information at the time of selection. In some studies [2,33,37,38], cross-validation was used to estimate the accuracy of genomic prediction when the data set was small or a specific focus was required. In the present study, a 4-fold cross-validation was carried out under two scenarios. The results showed that the accuracy of the genomic predictions in ValRandom was 0.114 higher than the predictions in ValFamily, averaged over the five traits in the analysis. This was because the animals in the test data set had half/full sibs in the training data in the ValRandom scenario, but not in ValFamily. The results were similar to those of previous studies on the effect of relatedness on the accuracy of genomic predictions. For example, the studies by Legarra et al. [39] and Kapell et al. [38] in mice, the study by Saatchi et al. [28] in beef cattle and the study by Gao et al. [23] in dairy cattle. These results indicated that the validation accuracy is greatly influenced by how the training and test data are created in the design of the validation study. An ideal validation procedure should be highly consistent with the real breeding scheme. However, it is often limited by the structure of the data in hand. It could be argued that the accuracy of GEBV is underestimated in ValFamily and overestimated in ValRandom, because in general sires (may be also dam) of candidates are in the reference population (not the case in ValFamity) while sibs are not in the reference population (the case in ValRandom). Considering the real scenario of chicken breeding, the true accuracy is expected to be between the accuracies in the two scenarios for the fives traits in the present population, based on training data of such a small size.

### Models for genomic predictions

A number of previous studies have been carried out that compared statistical models for genomic prediction. In general, simulation studies with the assumption that few QTLs account for a large proportion of the additive genetic variance have shown that the predictive ability of Bayesian variable shrinkage and variable selection models are superior to linear BLUP models [16,40,41]. However, studies in dairy cattle populations have reported that linear BLUP models perform as well as Bayesian models for most traits [4,5].

In the current study, GBLUP and the BayesLASSO produced genomic predictions of similar accuracies. None of these three models showed a consistently better performance for carcass traits; however, the differences among these models were very small (less than 0.01). BayesMix4 performed slightly better than the other models for growth traits. The results were similar to those obtained in other farm animals. For example, Ostersen et al. [32] compared a GBLUP model, a BayesLASSO model and a Bayesian mixture with two normal distributions, and found no differences between the three models in terms of the accuracy of genomic prediction in Duroc pigs. Gao et al. [23] compared models for genomic prediction in a Nordic Holstein population, and reported that GBLUP and the BayesLASSO had similar predictive abilities, and that BayesMix4 performed slightly better than the other models. The authors suggested that a mixture model with four normal distributions representing the prior distribution of SNP effects could better describe the distribution of true SNP effects than a mixture model with two normal distributions.

Some studies [42-44] reported that a model including a residual polygenic effect increased the accuracy of genomic prediction, because markers might not account for all the additive genetic variation. However, in mice, Legarra et al. [39] reported that a model including marker effects and polygenic effects had a poorer predictive ability than a model including marker effects only. The authors argued that polygenic genetic values and “marker-explained” global genetic values are expected to be very collinear, leading to poor quality estimation. In the present study, an additional analysis showed a genomic prediction model including polygenic effects did not improve the accuracy of the prediction (results not shown). This implied that the markers on the 60K chip accounted for almost all the additive genetic variation of the population.

## Conclusions

The results of this study show that in a breed with small effective population size, accurate GEBV can be obtained from a relatively small reference population. Moreover genomic prediction is a feasible approach for accurate selection in chicken breeding programs, especially for the traits which are difficult to be measured such as carcass traits.

## Methods

### Ethics statement

The Animal Care Committee of the Institute of Animal Science, Guangdong Academy of Agricultural Sciences (Guangzhou, People’s Republic of China) approved the current study (Approval No. GAAS-IAS-2009-73).

### Population and data

The birds in this study came from a population of a three-generation intercross between the “High Quality chicken LineA” (HQLA) and the Huiyang Beard chicken (HB), as described in Sheng et al. [45]. The HQLA line is a commercial line, which has been under selection for fast growth for more than 10 generations, while maintaining good meat quality. The HB line is a local Chinese breed, which is characterized by slow growth and high meat quality. In this study, 582 genotyped birds were used, comprising 20 F_0_ individuals, 51 F_1_ birds, and 511 F_2_ birds, which came from 8 half-sib families.

The birds were genotyped using the Illumina Chicken 60K SNP Beadchip [9] by DNA LandMarks Inc., Saint-Jean-sur-Richelieu, Canada. Deleting the SNP markers that had a call rate less than 95%, Gentrain scores less than 0.6, or a minor allele frequency less than 0.01 comprised the quality control. After this screening, a high quality set of 46,672 SNP markers was retained.

During the first 5 weeks of life, the birds were kept with group cages by hatch, and were provided with a starter feed (2,900 kcal of ME/kg and 200 g/kg of CP). From 6th to 13th week, all birds were housed in single cages, and provided with a grower feed (2,950 kcal of ME/kg and 180 g/kg of CP). The birds had free access to feed and water. The breeding facility supplied 24-hour lighting. A water curtain system was used to adjust the temperature.

Traits measured in the analysis included BW6, BW12, EP, BMP, and LMP. Table 5 shows the mean and standard deviation of the five traits for the 511 F_2_ animals.

**Table 5:** **Descriptions of the phenotypic data**

**Trait** ^**1**^	**No. of records**	**Mean**	**Standard deviation**
BW6	511	802.33	133.58
BW12	511	2027.06	360.27
EP	511	66.57	1.82
BMP	511	17.40	1.43
LMP	511	23.73	2.57

^1^ *BW*6 = body weight at 6th weeks; *BW*12 = body weight at 12th weeks; *EP* = eviscerating percentage; *BMP* = breast muscle percentage; *LMP* = leg muscle percentage.

### Statistical analysis

Conventional EBV was predicted using a BLUP model. Genomic breeding values were predicted using a GBLUP model, a Bayesian Lasso model and a Bayesian mixture model.

#### BLUP model

The BLUP model [46] to predict conventional EBV was:

$$ \mathrm{y} = \mathrm{X}\mathrm{b} + \mathrm{Z}\mathrm{a} + \mathrm{e} $$

where y is the vector of the observations, b is the vector of the fixed effect (sex and hatch), X is the incidence matrix of b, a is the vector of additive genetic effects of the birds in the pedigree, Z is the incidence matrix of a, and e is the vector of the residuals. It was assumed that a ~ N $\left(0,\mathrm{A}{\sigma}_a^2\right)$ and e ~ N $\left(0,\mathrm{I}{\sigma}_e^2\right)$, where A was a pedigree-based genetic relationship matrix and ${\sigma}_a^2$ was the additive genetic variance, and ${\sigma}_a^2$ is the residual variance.

#### GBLUP model

The GBLUP model [19] used to predict GEBV was as follows:

$$ \mathrm{y} = \mathrm{X}\mathrm{b} + \mathrm{Z}\mathrm{u} + \mathrm{e} $$

where the definitions of y, b, X, and e are the same as those in the BLUP model, u was the vector of additive genetic effects of genotyped individuals and Z was the incidence matrix of u. It was assumed that u ~ N $\left(0,\mathrm{G}{\sigma}_u^2\right)$, where G was the genomic relationship matrix constructed using SNP information [19] and ${\sigma}_a^2$ was the genomic additive genetic variance.

#### Bayesian LASSO model (BayesLASSO) and Bayesian mixture model (BayesMix4)

In the Bayesian analysis, the effects of SNPs were estimated using the following model:

$$ \mathrm{y} = \mathrm{X}\mathrm{b} + \mathrm{M}\mathrm{q} + \mathrm{e} $$

where q was the vector of random SNP effects, M was the matrix of genotype indicators, and y and e are the same as in the GBLUP model. The difference between BayesLASSO [18] and the BayesMix4 [23] is the assumption on the prior distribution of SNP effects. The BayesLASSO assumes that the effects of all SNPs follow a double exponential distribution.

$$ \mathrm{p}\left(\mathrm{q}\right)={\prod}_{j=1}^k\frac{1}{2}\lambda {e}^{-\lambda \left|{q}_j\right|} $$

where k was the number of markers, λ was a rate parameter that was sampled from a uniform distribution.

BayesMix4 assumes that the distribution of marker effects is a mixture of four normal distributions. In the current study, mixing proportions in this distribution were taken as known and set to π_1_ = 0.889, π_2_ = 0.1, π_3_ = 0.01 and π_4_ = 0.001, where π_1_ was the proportion of the distribution with the smallest variance, and π_4_ was the proportion of the distribution with the largest variance [23].

GEBV of individual i was defined as $GEB{V}_i={\displaystyle {\sum}_{j=1}^k{m}_{ij}{\widehat{q}}_j}$, where ${\widehat{q}}_j$ was the estimated effect of marker j, and m_ij_ was the genotype of marker j for individual i, and k was the number of markers. The DMU package [47] was used to analyze the BLUP and GBLUP models. Variance components were estimated implementing the average information restricted maximum likelihood algorithm [48], using a linear mixed model in the same form as the BLUP model. The Bayesian analysis was run with a single chain of 50,000 cycles. The first 20,000 cycles were discarded as the burn-in period, and each 20^th^ sample of the remaining 30,000 samples was saved for posterior analysis. The BayZ package (http://www.bayz.biz/) performed the analysis of the Bayesian models.

### Cross-validation

Two scenarios of 4-fold cross-validation were used to evaluate the accuracy of the genomic predictions. In the first scenario, the 8 half-sib families were divided into four subsets of similar sizes, i.e., a family sample, such that individuals in one subset did not have any half-sibs or full-sibs in the other subsets. In each fold of cross-validation, one of the four subsets was used as the test data, where the phenotypic values were masked during genomic prediction; the remaining three subsets were used as the training data. In the second scenario, 511 individuals were randomly split into four subsets, i.e., a random sample. As in the scenario of the family sample, in each fold of validation, one subset was used as the test data and the other three subsets were the training data. The numbers of birds in the test and training data sets for each fold of validation are shown in Table 6.

**Table 6:** **Number of birds in the training and test data sets of the 4**-**fold cross**-**validation in the scenarios of family sample and random sample**

**Data**	**Training data**	**Validating data**
Family_fold1	381	130
Family_fold2	383	128
Family_fold3	385	126
Family_fold4	384	127
Random_fold1	383	128
Random_fold2	383	128
Random_fold3	383	128
Random_fold4	384	127

The accuracy of GEBV was assessed by the correlation between GEBV and corrected phenotypic value (y_c_), where y_c_ was defined as the original phenotypic value corrected for fixed effects (sex and batch effects) which were estimated using the conventional BLUP model based on the full dataset, i.e., y_c_ = y – sex effect – batch effect.

Power analysis [49] was implemented to test the differences between correlation coefficient and zero at a level of Alpha =0.05. Power is the ability to find a statistically significant difference when the null hypothesis is in fact false, defined as 1-β, where β is the type II error rate. In this study Alpha (the Type I error rate) was set at 0.05 and power at 0.85 was accepted. As population size is increase, the power is also increase. To improve statistic power, the data pooled over the four folds of validations was used to calculate the correlation coefficients.

Paired t test [50] was used to compare these prediction models based on the correlation of each fold. The paired t test was performed by treating a fold as a subject and taking a pair of correlation coefficients for the fold from two models as a matched pair of observations. T value was calculated by follow formula, $\mathrm{t}=\frac{\overline{d}}{S_D/\sqrt{n}}$ where $\overline{d}$ is the mean of d over the 4 folds (d was defined as the difference of the correlation coefficients between two models in each fold) and ${S}_D$ is standard deviation of d.

Regression of y_c_ on GEBV was used to measure the lack of bias of GEBV. The regression would not differ significantly from one if GEBV was an unbiased estimate of the true breeding value [36]. The analyses are performed using R 2.15.0 package (http://www.r-project.org/).

## Additional file

Additional file 1: Table S1. Correlations between corrected phenotypic values and genomic predictions for the birds in the test sets.

## References

[1] Colombani C, Legarra A, Fritz S, Guillaume F, Croiseau P, Ducrocq V, Robert-Granié C (2013). Application of Bayesian least absolute shrinkage and selection operator (LASSO) and BayesCπ methods for genomic selection in French Holstein and Montbéliarde breeds. J Dairy Sci.

[2] Su G, Guldbrandtsen B, Gregersen VR, Lund MS (2010). Preliminary investigation on reliability of genomic estimated breeding values in the Danish Holstein population. J Dairy Sci.

[3] VanRaden PM, Sullivan PG (2010). International genomic evaluation methods for dairy cattle. Genet Sel Evol.

[4] Hayes BJ, Bowman PJ, Chamberlain AJ, Goddard ME (2009). Invited review: genomic selection in dairy cattle: progress and challenges. J Dairy Sci.

[5] VanRaden PM, Van Tassell CP, Wiggans GR, Sonstegard TS, Schnabel RD, Taylor JF, Schenkel FS (2009). Invited Review: Reliability of genomic predictions for North American Holstein bulls. J Dairy Sci.

[6] Tribout T, Larzul C, Phocas F (2012). Efficiency of genomic selection in a purebred pig male line. J Anim Sci.

[7] Christensen OF, Madsen P, Nielsen B, Ostersen T, Su G (2012). Single-step methods for genomic evaluation in pigs. Animal.

[8] Duchemin S, Colombani C, Legarra A, Baloche G, Larroque H, Astruc J, Barillet F, Robert-Granie C, Manfredi E (2012). Genomic selection in the French Lacaune dairy sheep breed. J Dairy Sci.

[9] Groenen M, Megens H-J, Zare Y, Warren W, Hillier L, Crooijmans R, Vereijken A, Okimoto R, Muir W, Cheng H (2011). The development and characterization of a 60K SNP chip for chicken. BMC Genomics.

[10] Schaeffer LR (2006). Strategy for applying genome-wide selection in dairy cattle. J Anim Breed Genet.

[11] González-Recio O, Gianola D, Long N, Weigel KA, Rosa GJM, Avendaño S (2008). Nonparametric methods for incorporating genomic information into genetic evaluations: an application to mortality in broilers. Genetics.

[12] González-Recio O, Gianola D, Rosa GJM, Weigel KA, Kranis A (2009). Genome-assisted prediction of a quantitative trait measured in parents and progeny: application to food conversion rate in chickens. Genet Sel Evol.

[13] Chen CY, Misztal I, Aguilar I, Tsuruta S, Meuwissen THE, Aggrey SE, Wing T, Muir WM (2011). Genome-wide marker-assisted selection combining all pedigree phenotypic information with genotypic data in one step: an example using broiler chickens. J Anim Sci.

[14] Wei M, van der Werf JH (1995). Genetic correlation and heritabilities for purebred and crossbred performance in poultry egg production traits. J Anim Sci.

[15] Moyer SE, Collins WM, Skoglund WC (1962). Heritability of body weight at three ages in cross-bred broiler chickens resulting from two systems of breeding. Poult Sci.

[16] Meuwissen THE, Hayes BJ, Goddard ME (2001). Prediction of total genetic value using genome-wide dense marker maps. Genetics.

[17] Habier D, Fernando RL, Dekkers JCM (2007). The impact of genetic relationship information on genome-assisted breeding values. Genetics.

[18] de los Campos G, Naya H, Gianola D, Crossa J, Legarra A, Manfredi E, Weigel K, Cotes JM (2009). Predicting quantitative traits with regression models for dense molecular markers and pedigree. Genetics.

[19] VanRaden PM (2008). Efficient methods to compute genomic predictions. J Dairy Sci.

[20] Gianola D (2006). Genomic-assisted prediction of genetic value with semiparametric procedures. Genetics.

[21] Su G, Christensen OF, Ostersen T, Henryon M, Lund MS (2012). Estimating additive and non-additive genetic variances and predicting genetic merits using genome-wide dense single nucleotide polymorphism markers. PLoS One.

[22] Meuwissen THE (2009). Accuracy of breeding values of ’unrelatedz’ individuals predicted by dense SNP genotyping. Genet Sel Evol.

[23] Gao H, Su G, Janss L, Zhang Y, Lund MS (2013). Model comparison on genomic predictions using high-density markers for different groups of bulls in the Nordic Holstein population. J Dairy Sci.

[24] Goddard M (2009). Genomic selection: prediction of accuracy and maximisation of long term response. Genetica.

[25] Daetwyler HD, Villanueva B, Woolliams JA (2008). Accuracy of predicting the genetic risk of disease using a genome-wide approach. PLoS One.

[26] HAYES BJ, VISSCHER PM, GODDARD ME (2009). Increased accuracy of artificial selection by using the realized relationship matrix. Genet Res.

[27] Lund M, de Roos A, de Vries A, Druet T, Ducrocq V, Fritz S, Guillaume F, Guldbrandtsen B, Liu Z, Reents R, Schrooten C, Seefried F, Su G (2011). A common reference population from four European Holstein populations increases reliability of genomic predictions. Genet Sel Evol.

[28] Saatchi M, McClure M, McKay S, Rolf M, Kim J, Decker J, Taxis T, Chapple R, Ramey H, Northcutt S, Bauck S, Woodward B, Dekkers J, Fernando R, Schnabel R, Garrick D, Taylor J (2011). Accuracies of genomic breeding values in American Angus beef cattle using K-means clustering for cross-validation. Genet Sel Evol.

[29] Saatchi M, Schnabel R, Rolf M, Taylor J, Garrick D (2012). Accuracy of direct genomic breeding values for nationally evaluated traits in US Limousin and Simmental beef cattle. Genet Sel Evol.

[30] Snelling WM, Allan MF, Keele JW, Kuehn LA, Thallman RM, Bennett GL, Ferrell CL, Jenkins TG, Freetly HC, Nielsen MK, Rolfe KM (2011). Partial-genome evaluation of postweaning feed intake and efficiency of crossbred beef cattle. J Anim Sci.

[31] Lillehammer M, Meuwissen THE, Sonesson AK (2011). Genomic selection for maternal traits in pigs. J Anim Sci.

[32] Ostersen T, Christensen O, Henryon M, Nielsen B, Su G, Madsen P (2011). Deregressed EBV as the response variable yield more reliable genomic predictions than traditional EBV in pure-bred pigs. Genet Sel Evol.

[33] Daetwyler H, Swan A, van der Werf J, Hayes B (2012). Accuracy of pedigree and genomic predictions of carcass and novel meat quality traits in multi-breed sheep data assessed by cross-validation. Genet Sel Evol.

[34] Wolc A, Stricker C, Arango J, Settar P, Fulton J, O’Sullivan N, Preisinger R, Habier D, Fernando R, Garrick D, Lamont S, Dekkers J (2011). Breeding value prediction for production traits in layer chickens using pedigree or genomic relationships in a reduced animal model. Genet Sel Evol.

[35] Su G, Madsen P, Nielsen U, Mantysaari E, Aamand G, Christensen O, Lund M (2012). Genomic prediction for Nordic Red Cattle using one-step and selection index blending. J Dairy Sci.

[36] Su G, Brondum RF, Ma P, Guldbrandtsen B, Aamand GR, Lund MS (2012). Comparison of genomic predictions using medium-density (similar to 54,000) and high-density (similar to 777,000) single nucleotide polymorphism marker panels in Nordic Holstein and Red Dairy Cattle populations. J Dairy Sci.

[37] Luan T, Woolliams JA, Lien S, Kent M, Svendsen M, Meuwissen THE (2009). The accuracy of Genomic Selection in Norwegian red cattle assessed by cross-validation. Genetics.

[38] Kapell D, Sorensen D, Su G, Janss L, Ashworth C, Roehe R (2012). Efficiency of genomic selection using Bayesian multimarker models for traits selected to reflect a wide range of heritabilities and frequencies of detected quantitative traits loci in mice. BMC Genet.

[39] Legarra A, Robert-Granie C, Manfredi E, Elsen JM (2008). Performance of genomic selection in mice. Genetics.

[40] Lund M, Sahana G, de Koning D-J, Su G, Carlborg O (2009). Comparison of analyses of the QTLMAS XII common dataset. I: Genomic selection. BMC Proceedings.

[41] Guo G, Lund MS, Zhang Y, Su G (2010). Comparison between genomic predictions using daughter yield deviation and conventional estimated breeding value as response variables. J Anim Breed Genet.

[42] Calus MPL, Veerkamp RF (2007). Accuracy of breeding values when using and ignoring the polygenic effect in genomic breeding value estimation with a marker density of one SNP per cM. J Anim Breed Genet.

[43] Gao H, Christensen O, Madsen P, Nielsen U, Zhang Y, Lund M, Su G (2012). Comparison on genomic predictions using three GBLUP methods and two single-step blending methods in the Nordic Holstein population. Genet Sel Evol.

[44] Mrode R, Moore K, Winters M, Coffey M (2012). Evaluating the impact of including residual polygenic effects in dairy genomic evaluations using Bayesian methods. Interbull Bulletin.

[45] Sheng Z, Pettersson M, Hu X, Luo C, Qu H, Shu D, Shen X, Carlborg O, Li N (2013). Genetic dissection of growth traits in a Chinese indigenous x commercial broiler chicken cross. BMC Genomics.

[46] Henderson CR (1975). Best linear unbiased estimation and prediction under a selection model. Biometrics.

[47] Madsen P, Su G, Labouriau R, Christensen OF (2010). DMU—A package for Analyzing Multivariate Mixed Models.

[48] Gilmour AR, Thompson R, Cullis BR (1995). Average information REML: an efficient algorithm for variance parameter estimation in linear mixed models. Biometrics.

[49] Cohen J (1988). Statistical Power Analysis for the Behavioral Sciences.

[50] Elston RC, Johnson W (2008). Basic Biostatistics for Geneticists and Epidemiologists: A Practical Approach.

